# The impact on clinical outcomes after 1 year of implementation of an artificial intelligence solution for the detection of intracranial hemorrhage

**DOI:** 10.1186/s12245-023-00523-y

**Published:** 2023-08-11

**Authors:** Dmitry Kotovich, Gilad Twig, Zeev Itsekson-Hayosh, Maximiliano Klug, Asaf Ben Simon, Gal Yaniv, Eli Konen, Noam Tau, Daniel Raskin, Paul J. Chang, David Orion

**Affiliations:** 1 The Institute for Research in Military Medicine, The Faculty of Medicine, The Hebrew University of Jerusalem, Tel Aviv, Israel; 2 The IDF Medical Corps, 9112102 Tel Aviv, Israel; 3 Center of Stroke and Neurovascular Disorders, Sheba Medical Center, Tel HaShomer, Ramat Gan, affiliated to Sackler Faculty of Medicine, Tel Aviv University, 52621 Tel Aviv, Israel; 4 Department of Diagnostic Imaging, Sheba Medical Center, Tel HaShomer, Ramat Gan, Israel, affiliated to Sackler Faculty of Medicine, Tel Aviv University, 52621 Tel Aviv, Israel; 5 Sackler School of Medicine, Faculty of Medicine, Tel Aviv University, 69978 Tel Aviv, Israel; 6 Department of Radiology, University of Chicago Medical Center, Chicago, Illinois 60637 USA; 7 Center of Stroke and Neurovascular Disorders, Sheba Medical Center, Tel HaShomer, Ramat Gan, affiliated to Sackler Faculty of Medicine, Tel Aviv University, 52621 Tel Aviv, Israel

**Keywords:** Artificial intelligence, Intracranial hemorrhage, Modified ranking scale

## Abstract

**Background:**

To assess the effect of a commercial artificial intelligence (AI) solution implementation in the emergency department on clinical outcomes in a single level 1 trauma center.

**Methods:**

A retrospective cohort study for two time periods—pre-AI (1.1.2017–1.1.2018) and post-AI (1.1.2019–1.1.2020)—in a level 1 trauma center was performed. The ICH algorithm was applied to 587 consecutive patients with a confirmed diagnosis of ICH on head CT upon admission to the emergency department.

Study variables included demographics, patient outcomes, and imaging data. Participants admitted to the emergency department during the same time periods for other acute diagnoses (ischemic stroke (IS) and myocardial infarction (MI)) served as control groups. Primary outcomes were 30- and 120-day all-cause mortality. The secondary outcome was morbidity based on Modified Rankin Scale for Neurologic Disability (mRS) at discharge.

**Results:**

Five hundred eighty-seven participants (289 pre-AI—age 71 ± 1, 169 men; 298 post-AI—age 69 ± 1, 187 men) with ICH were eligible for the analyzed period. Demographics, comorbidities, Emergency Severity Score, type of ICH, and length of stay were not significantly different between the two time periods. The 30- and 120-day all-cause mortality were significantly reduced in the post-AI group when compared to the pre-AI group (27.7% vs 17.5%; *p* = 0.004 and 31.8% vs 21.7%; *p* = 0.017, respectively). Modified Rankin Scale (mRS) at discharge was significantly reduced post-AI implementation (3.2 vs 2.8; *p* = 0.044).

**Conclusion:**

The added value of this study emphasizes the introduction of artificial intelligence (AI) computer-aided triage and prioritization software in an emergent care setting that demonstrated a significant reduction in a 30- and 120-day all-cause mortality and morbidity for patients diagnosed with intracranial hemorrhage (ICH). Along with mortality rates, the AI software was associated with a significant reduction in the Modified Ranking Scale (mRs).

**Supplementary Information:**

The online version contains supplementary material available at 10.1186/s12245-023-00523-y.

## Key points

- The 30-day and 120-day post-discharge all-cause mortality was observed to be significantly lower in the post-artificial intelligence (post-AI) implementation period compared to pre-AI period (27.7% vs 17.5% *p* = 0.004; 31.8% vs 21.7% *p* = 0.017, respectively).
- Modified Rankin Scale (mRS) for neurological disability at discharge was significantly reduced post-AI implementation (3.2 vs 2.8; *p* = 0.044).

**Supplementary Information:**

The online version contains supplementary material available at 10.1186/s12245-023-00523-y.

## Background

Artificial intelligence (AI) in healthcare is growing rapidly. In radiology, AI has the potential to transform the healthcare field by integrating into the radiology workflow and improving the efficiency and efficacy of medical imaging. Many studies have been published on the potential of AI to improve the triage, prioritization, and detection of critical conditions and pathologies. Intracranial hemorrhage is one condition that is highly impacted by AI’s ability in prioritizing and triaging suspected findings, thus leading to earlier therapeutic interventions. Literature has shown that earlier detection and initiation of interventions can reduce the risk of complications and overall mortality and morbidity for ICH patients. With regard to the potential of AI, a few recent studies have shown a significant impact of AI on clinical outcomes for ICH patients. One most recent study showed a significant reduction in hospital length of stay after the implementation of AI software into the radiological workflow at a large academic center. To date, there have been no studies looking at AI’s impact on all-cause mortality rates for ICH patients in the emergent care setting.

A background literature search was conducted using PubMed and Open Access journal sources. Observational studies as well as systematic reviews and metanalysis were reviewed.

The added value of this study emphasizes the introduction of artificial intelligence (AI) computer-aided triage and prioritization software in an emergent care setting that demonstrated a significant reduction in 30- and 120-day all-cause mortality and morbidity for patients diagnosed with intracranial hemorrhage (ICH). Along with mortality rates, the AI software was associated with a significant reduction in the Modified Ranking Scale (mRs).

The study highlights the potential of AI-triage software to offer a new path toward impacting mRs score and mortality rates in this critical population.

## Introduction

Intracerebral hemorrhage (ICH) is a critical condition with high mortality and morbidity rates [1]. The 30-day mortality rate ranges between 40 and 50%, with about half of the deaths occurring within the first 24 h [2, 3]. Studies have shown early detection of ICH is critical in improving treatment outcomes, thus improving patient outcomes and comorbidities [4–6]. Rapid detection and appropriate therapeutic interventions have the potential to reduce the overall mortality rate associated with ICH [7, 8].

AI algorithms have evolved greatly in the past years, especially on image-recognition tasks, creating a myriad of applications in the medical image analysis field, propelling it forward at a rapid pace [9, 10]. Recent studies have shown that AI algorithms have enhanced physician’s clinical workflow, mainly by automated pathology detection [11–15] on top of verbal communication, thus improving therapeutic interventions. Prior studies done on AI and ICH have showed a reduction in turn-around time [16, 17] with high specificity and sensitivity [18]. Improving care management and accelerating treatment strategies through AI software have shown to improve patient safety and outcomes [19, 20].

This study aims to explore the impact of using an AI solution in radiology for computer-aided triage and prioritization on patient and clinical outcomes in the ICH population.

## Materials and methods

### Study design and oversight

**Fig. 1:**
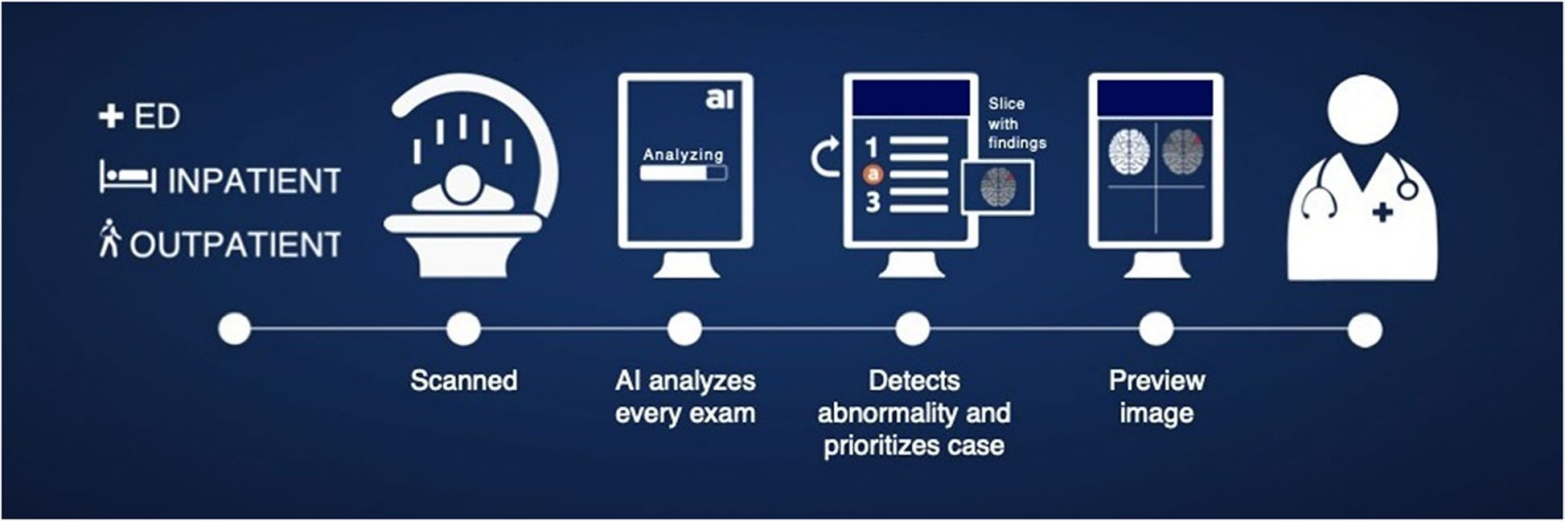
AI worfklow. A representation of the radiologist workflow with the artificial intelligence software implementation

The study was conducted as a retrospective non-randomized cohort study using an FDA-approved, AI-based computer-aided triage, and prioritization solution (Aidoc, Tel Aviv, Israel) approved for the detection of all types of intracranial hemorrhages in radiology. Prior to the AI solution implementation (pre-AI), the radiologist worklist would be reviewed according to a FIFO (first in, first out) methodology. The AI solution was implemented at our emergency department institution between February 2018 and September 2018. The system operates as follows: all relevant CT studies are automatically sent for AI analysis with no manual trigger. Upon detection of suspected positive ICH findings, the AI solution delivers notifications directly to the radiologist workstation. The system was declared workflow-integrated and clinically ready once all the CT scanners and radiologist workstations were integrated. After the implementation, the radiologist worklist would receive notifications for scans with suspected positive ICH findings according to the AI algorithm, thus changing from the FIFO methodology to an urgency-cased methodology (post-AI). The description of the solution workflow is shown in Fig. 1.

The study was IRB approved with approval number 3532–16-SMC from September 7, 2020.

### Data acquisition

Data was retrieved from the Chameleon^©^ electronic health record (EHR) system in our hospital using the MDClone software—a data extraction and synthetization platform that provides patient-level index-organized event data (http://www.mdclone.com).

Data was extracted between the timeframes 1.1.2017–1.1.2018 (pre-AI) and 1.1.2019–1.1.2020 (post-AI). The AI solution was fully deployed in all the emergency department reading rooms served by the radiologists on call.

### Study participants

All patients 18 years and older that were admitted for the first time to our institution’s emergency department who underwent a non–contrast head computed tomography scan during the emergency department admission with a positive finding of any ICH type (subdural, epidural, subarachnoid, intraparenchymal, and intraventricular hemorrhage) on finalized radiologist report were included in the study population. The study population was split into two groups for the pre-AI and post-AI periods, respectively. Two additional comparable pathologies were included as controls: ischemic stroke (IS) and myocardial infarction (MI) during the same time periods. These served as internal controls to equalize the possible differences in healthcare protocols between the pre-AI and post-AI time periods. Both control groups included participants older than 18 years admitted for the first time to our institution’s emergency department. Ischemic stroke was defined as positive ischemic findings using computed tomography after a finalized radiologist report. Myocardial infarction was defined as a positive ischemic finding using troponin level change over time with supportive evidence (typical symptoms, suggestive electrocardiographic changes, imaging evidence of new myocardium abnormality). All included participants were discharged with the same primary diagnosis on admission. Acquisition and analysis of all the data described in Table 1 were done up to May 1, 2020, to enable a 120-day mortality analysis of participants admitted to the emergency department by December 31, 2019.

**Table 1:** Demographic, comorbidity, medications, and clinical metrics in the ICH, ischemic stroke, and myocardial infarction datasets

Measure	ICH		*P* value	Ischemic stroke		*P* value	Myocardial infarction		*P* value
	Pre-AI (*N* = 289)	Post-AI (*N* = 298)		Pre-AI (*N* = 608)	Post-AI (*N* = 1126)		Pre-AI (*N* = 217)	Post-AI (*N* = 160)	
**Age, year mean (SE)**	71.1 (0.88)	68.7 (0.95)	0.062	71.3 (0.52)	71.2 (0.4)	0.78	65.4 (0.89)	66.4 (1.1)	0.5
**Man, % (SE%)**	58.5% (2.9%)	62.8% (2.8%)	0.32	53.4% (2%)	54.5% (1.4%)	0.71	80.6% (2.7%)	69.3% (3.6%)	0.02
**Smokers, % (SE%)**	5.8% (1.4%)	5.7% (1.3%)	1	5.2% (0.9%)	7% (0.7%)	0.18	9.6% (2%)	11.8% (2.5%)	0.61
**Diabetes Mellitus, % (SE%)**	15.2% (2.1%)	12.7% (2%)	0.45	17.7% (1.5%)	20.7% (1.2%)	0.15	20.7% (2.7%)	17.5% (3%)	0.5
**Hypertension, % (SE%)**	26.9% (2.6%)	26.9% (2.6%)	0.37	36% (1.9%)	34.5% (1.4%)	0.57	30.8% (3.1%)	26.8% (3.5%)	0.46
**Chronic heart failure, % (SE%)**	4.5% (1.2%)	4.5% (1.2%)	0.76	5.9% (1%)	4.8% (0.6%)	0.42	4.6% (1.4%)	5.6% (1.8%)	0.83
**Anticoagulation and** **anti-aggregation agents, % (SE%)**	41.8% (3.0%)	28.8% (2.6%)	0.001	53.4% (2.0%)	35.2% (1.4%)	< 0.001	52.1% (3.4%)	28.7% (3.6%)	< 0.001
**Hypertensive agents, % (SE%)**	8.3% (1.6%)	6.1% (1.4%)	0.36	5.4% (0.9%)	4.5% (0.6%)	0.47	4.1% (1.3%)	1.8% (1.0%)	0.34
**White blood count median (IQR)**	9.6 (7.5–12.6)	9.9 (7.8–12.9)	0.42	8.2 (6.7–9.9)	7.9 (6.5–9.8)	0.22	11.1 (8.9–13.8)	10.9 (9.1–13.6)	0.58
**Hemoglobin count median (IQR)**	12.9 (11.8–14.3)	13.3 (12.1–14.7)	0.007	13.4 (12.3–14.4)	13.4 (12.4–14.5)	0.37	14.2 (12.8–15.3)	14.2 (13–15.3)	0.76
**Platelet count median (IQR)**	216 (179–263)	230 (190–276)	0.009	226 (188–264)	228 (188–271)	0.28	233 (194–277)	254 (207–260)	0.01
**CT-TAT**	8.24 (7.41)	7.2 (6.49)	0.07	–-		–-	–-		–-
**Emergency Severity Index 103, % (SE%)**	54.4% (2.9%)	61.7% (2.8%)	0.13	81.4% (1.5%)	78.7% (1.2%)	0.12	44.7% (3.3%)	60.0% (3.8%)	0.57
**Length of stay, days mean (SE)**	19.5 (2.8)	18.0 (1.7)	0.193	6.9 (0.7)	8.6 (0.54)	0.002	6.2 (0.54)	5.4 (0.51)	0.221
**Mortality after 30 days, % (SE%)**	27.7% (2.9%)	17.5% (2.3%)	0.004	2.4% (0.06%)	3.6% (0.5%)	0.33	7.0% (1.8%)	11.0% (2.5%)	0.550
**Mortality after 30 days, % change**	− 36.8%			50%			57.1%		
**Mortality after 120 days, % (SE%)**	31.8% (2.9%)	21.7% (2.4%)	0.017	4.9% (0.9%)	6.0% (0.7%)	0.50	8.0% (1.9%)	14.5% (2.8%)	0.170
**Mortality after 120 days, % change**	− 31.7%			22.4%			81.2%		
**Modified Ranking Scale baseline**	0.76 (1.309)	0.77 (1.243)	0.944	–-		–-	–-		–-
**Modified Ranking Scale Discharge**	3.17 (2.193)	2.84 (1.911)	0.044	–-		–-	–-		–-
**Modified Ranking Scale difference discharge, baseline**	2.37 (2.219)	2.03 (1.951)	0.041	–-		–-	–-		–-

*SE* standard error, *IQR* interquartile range

The confounding variables as well as clinical measures examined are shown in Table 1. Confounding variables included in the analysis were based on demographics, comorbidities, and medications: age, gender, smoking status, and complete blood count for white blood cells, hemoglobin, and platelet count, diabetes mellitus, hypertension, congestive heart failure, current use of anti-hypertensive agents, and anticoagulation and anti-aggregation agents (Table 3 in Supplements).

Emergency Severity Index (ESI) [21] was used to evaluate the participants’ clinical severity and therefore the emergency department resource utilization. An ESI score of 103 represents a median score (range of 101–105), and its percentile was used to compare the pre-AI and post-AI time periods.

Modified Rankin Scale for neurologic disability (mRS) was used for morbidity evaluation and was evaluated at admission (baseline) and discharge by a stroke neurologist according to standardized guidelines [21, 22].

Mortality was drawn from the hospital medical records software which is continuously updated by the Ministry of Health national mortality records.

Length of stay (LoS) was defined as the time (in days) from patient’s admission to the emergency room until complete discharge from the hospital or death.

Modified Rankin Scale was evaluated at admission—prior to current ICH—and at discharge from the hospital.

### Statistical analysis

The continuous variables are presented as means and standard errors or medians and interquartile ranges. Categorical variables are presented as percentages.

Statistical tests such as the independent *t* test, Wilcoxon-Mann–Whitney, and chi-square were performed to account for differences between the pre-AI and post-AI cohorts.

We used a multiple logistic regression model to analyze the dichotomous variables while controlling for confounding covariates. Odds ratios are presented to indicate the likelihood of these clinical measures occurring between the time frames.

A two-sided *p* value of less than 0.05 was used to indicate statistical significance. Results are reported with 95% confidence intervals (CI).

All analyses were performed with R software, version 3.6.3.

## Results

The ICH dataset included a total of 587 participants with confirmed ICH that underwent a computed tomography scan in their first emergency department visit, 289 for the pre-AI group and 298 for the post-AI group. The ischemic stroke dataset included 1734 participants who were admitted to the emergency department with a confirmed diagnosis, 608 for the pre-AI group and 1126 for the post-AI group. The myocardial infraction dataset included 377 participants who were admitted to the emergency department with a confirmed diagnosis, 217 for the pre-AI group and 160 for the post-AI group. No significant difference was found between the different ICH type rates and the time periods (*p* = 0.1339) (Table 1 in Supplements). The number of missing data and percentage out of the total in ICH, ischemic stroke, and myocardial infarction datasets were similar (Table 2 in Supplements).

### Demographics and comorbidities

Table 1 shows the complete comparison between the pre-AI group and the post-AI group for ICH, ischemic stroke (IS), and myocardial infarction (MI) demographic, comorbidity, medications, and clinical metrics—emergency severity index, length of stay, mortality at 30 and 12 days, and morbidity represented using Modified Ranking Scale.

No significant difference was observed for age, gender, smoking, diabetes mellitus, hypertension, chronic heart failure, or antihypertensive agent use for the ICH group (*p* = 0.062; *p* = 0.32, *p* = 1, *p* = 0.45, *p* = 0.37, *p* = 0.76, *p* = 0.36, respectively) or in any of the control groups of ischemic stroke and myocardial infarction (excluding *p* = 0.02 the male gender comparison between the pre-AI group and the post-AI group).

A significant difference was observed between the anticoagulants and anti-aggregant agents use in the pre-AI group compared to the post-AI group (41.8% vs 28.8%; *p* = 0.001) for the ICH dataset, which was constant for ischemic stroke and myocardial infarction datasets as well (*p* =  < 0.001 for both) (Table 3 in Supplements).

A significant difference was observed between the hemoglobin levels and platelet counts between the pre-AI group and post-AI group for the ICH dataset (12.9 vs 13.3; *p* = 0.007 and 216 vs 230; *p* = 0.009, respectively).

### Mortality

A significant decrease in the 30-day mortality rate was observed in the post-AI group compared to the pre-AI group (pre-AI 27.7% vs post-AI 17.5%, odds ratio = 0.48, CI of odd 0.29 to 0.79, *p* = 0.004), and a significant decrease in the 120-day mortality in the post-AI group in comparison to the pre-AI group (pre-AI 31.8% vs. post-AI 21.7%, odds ratio 0.58, CI of odds 0.37 to 0.91, *p* = 0.017) was observed for the ICH dataset.

**Fig. 2:**
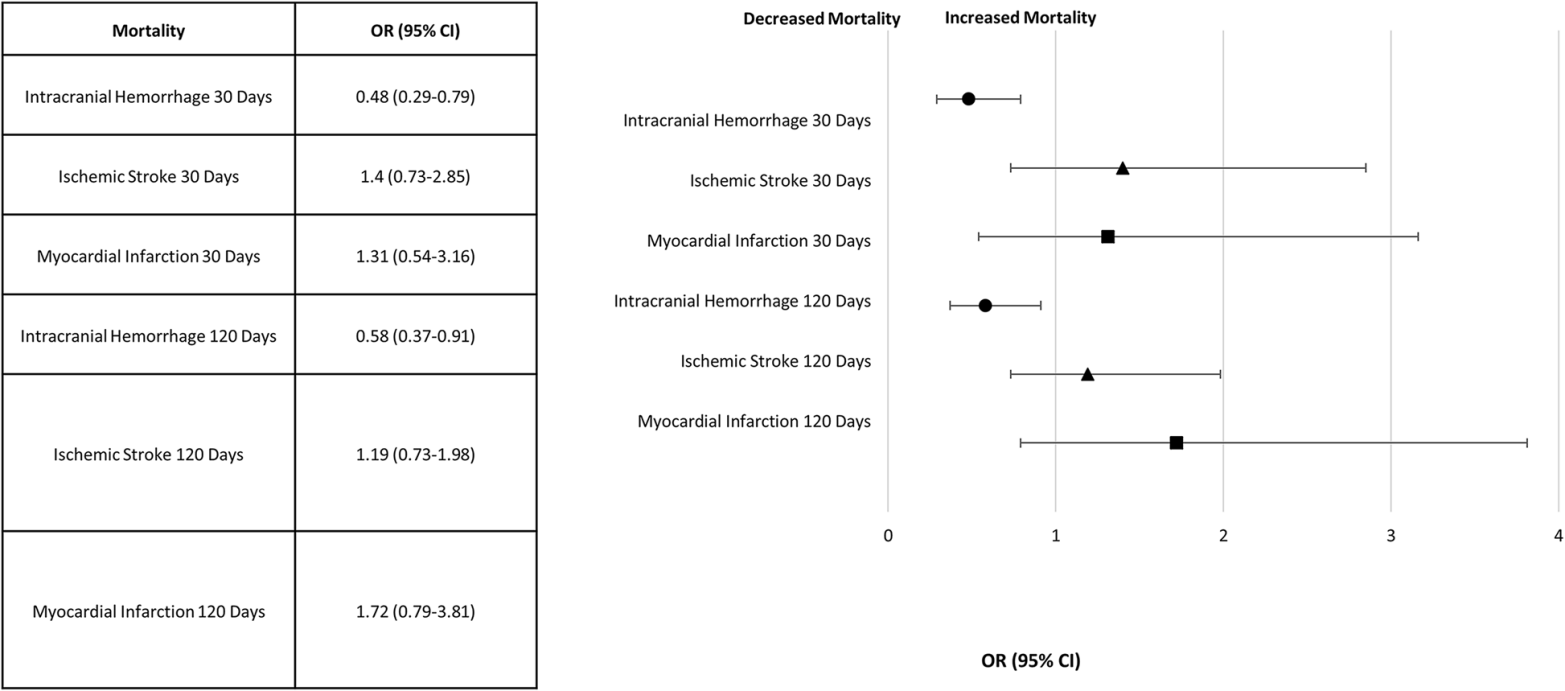
Forest plot for the odds ratio (OR) for mortality (30 and 120 days) after the artificial intelligence (AI) software implementation (post-AI) and the control groups (myocardial infarct (MI), ischemic stroke (IS)) mortality on the same time period

No similar difference was observed for the ischemic stroke dataset (30 days odds ratio 1.4 [CI 0.73–2.85] *p* = 0.33; 120-day odds ratio 1.19 [CI 0.73–1.98] *p* = 0.5) or myocardial infarction dataset (30 days odds ratio 1.31 [CI 0.54–3.16] *p* = 0.55; 120-day odds ratio 1.72 [CI 0.79–3.81] *p* = 0.17) after controlling for significant confounders. The forest plot for mortality OR is shown in Fig. 2.

### Anticoagulation and anti-aggregation usage and mortality for ICH dataset

Sub-analysis of the participants with and without anticoagulation and anti-aggregation treatment was performed between the pre-AI group and post-AI group and its effect on 30- and 120-day mortality (Table 2).

**Table 2:** Anticoagulation and anti-aggregation treatment and mortality for the ICH dataset

	**Under anticoagulation/anti-aggregation**		**Not under anticoagulation/anti-aggregation**	
	Not deceased	Deceased	Not deceased	Deceased
**Mortality 30 days**				
**Pre-AI,** ***N*** **(% of total)**	54 (63.5%)	31 (36.5%)	111 (77.6%)	32 (22.4%)
**Post-AI,** ***N*** **(% of total)**	62 (82.6%)	13 (17.4%)	165 (82.5%)	35 (17.5%)
**% Change**	− 52.3% *p* = 0.01147		− 21.8% *p* = 0.3245	
**Mortality 120 days**				
**Pre-AI,** ***N*** **(% of total)**	54 (58.1%)	39 (41.9%)	111 (74.5%)	38 (25.5%)
**Post-AI,** ***N*** **(% of total)**	62 (76.5%)	19 (23.5%)	165 (78.9%)	44 (21.1%)
**% Change**	− 43.9% *p* = 0.01561		− 17.2% *p* = 0.3897	

Participants with mortality above 30 days post-admission were removed from the analysis for the 30-day mortality (pre-AI 228/289 participants’ analysis; post-AI 275/298 participants’ analysis).

Participants with mortality above 120 days post-admission were removed from the analysis for the 120-day mortality (pre-AI 252/289 participants’ analysis; post-AI 290/298 participants’ analysis).

Both groups showed a decrease in mortality percentage between pre-AI and post-AI, for both the 30- and 120-day mortality, with a significant decrease for the subgroup under anticoagulation and anti-aggregation treatment (− 52.3% *p* = 0.0114 for the 30-day mortality and − 43.9% *p* = 0.0156 for the 120-day mortality).

### Modified Rankin Scale (mRS) comparison for ICH dataset

**Fig. 3:**
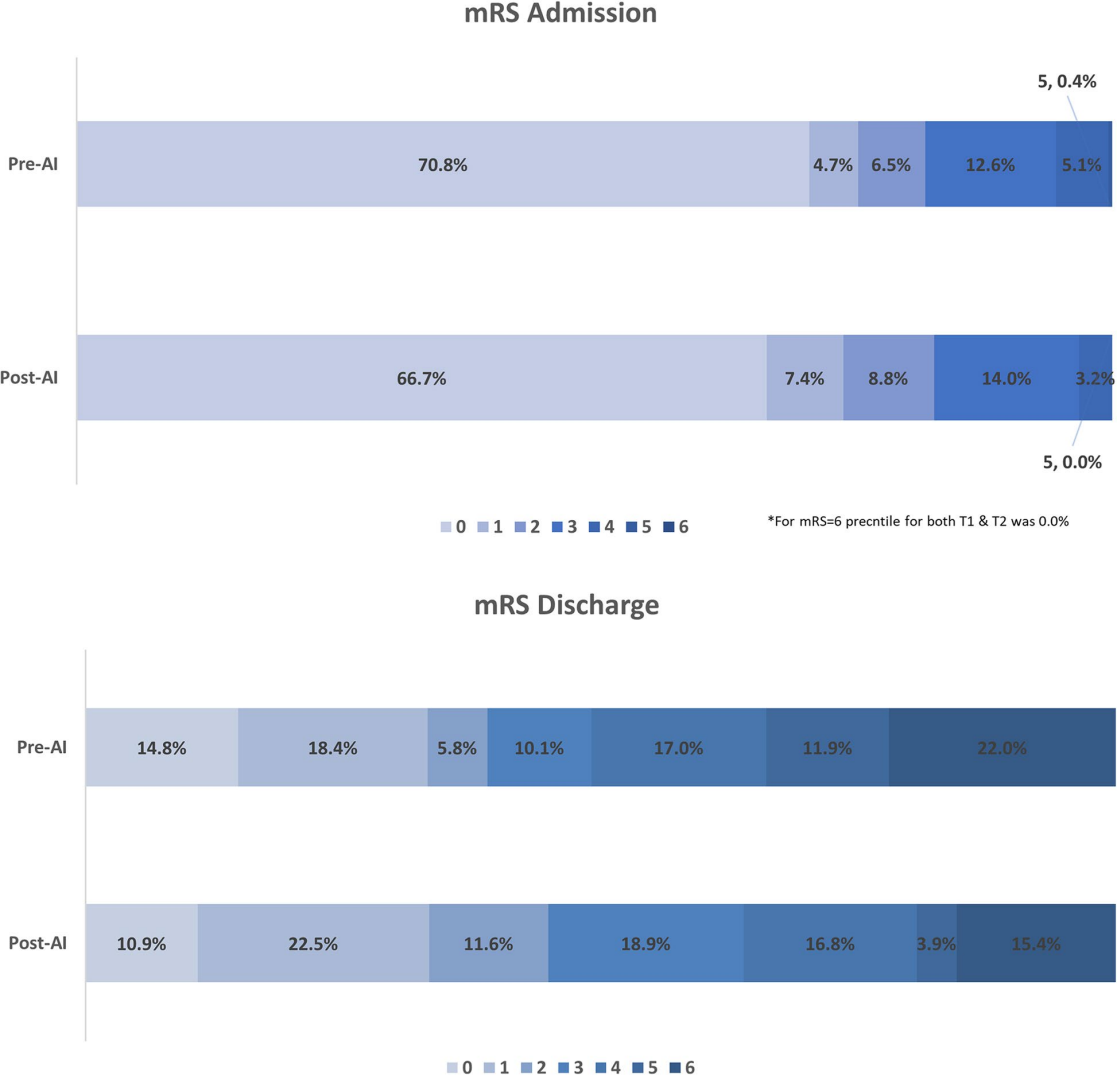
Modified Ranking Scale (mRS) value distribution at admission and discharge before (pre-AI) and after (post-AI) artificial intelligence (AI) software implementation. A higher mRS score means higher morbidity

There was no significant difference between the baseline score at admission in the pre-AI group and post-AI group (0.77 vs 0.77, *p* = 0.94). Modified Rankin Scale at discharge showed a significant difference between the time periods with a decrease in the post-AI group (3.17 vs 2.84, *p* = 0.044) (Fig. 3). Modified Rankin Scale difference between discharge and admission, for each group separately, showed a significant difference between the pre-AI group and post-AI group (2.37 vs 2.03; *p* = 0.041).

## Discussion

We compared two 1-year time periods, before and after AI implementation for intracranial hemorrhage (ICH) diagnosis in the emergency department and validated the results using a comparison to two other control pathologies. The two control pathologies—ischemic stroke and myocardial infarction—a common ischemic pathologies with similar risk factors and urgency for treatment, were not analyzed by the AI solution. No statistical difference between the two time periods was observed for demographics, comorbidities, emergency severity index [21], length of stay, and ICH type. A significant decrease in all-cause mortality after 30 and 120 days post-discharge with over 30% relative decrease in absolute mortality rate in the post-AI group for the ICH dataset was observed. Morbidity from ICH was evaluated using the Modified Rankin Scale (mRS) [22] for neurological disability. A significant difference in mRS score at discharge between the pre-AI and post-AI groups was observed, which resulted in a significantly lower mRS score difference between admission to discharge for the post-AI group.

Next, we aimed to try to understand the underlying mechanism of this observed reduction in mortality. A sub-analysis was performed between participants on anticoagulation or antiplatelet medications in comparison to those without. A significant decrease in mortality in the ICH dataset was shown for the subgroup under anticoagulation and anti-aggregation treatment. No significant difference in mortality was observed in the population who were not receiving anti-aggregation or anticoagulation medications before admission to the emergency department. The possibility of this finding may suggest that the fact that patients who are taking anti-aggregation or anticoagulation medications and have ICH can potentially benefit from earlier initiation of ICH treatments compared to patients who are not receiving treatment. Few studies have shown early initiation of ICH treatments may lead to improved clinical outcomes including a lower rate of neurological deterioration and hematoma expansion in this population [23–25]. A recent study showed that early initiation of ICH treatment reduced median intensive care unit (ICU) LoS and cost of hospitalization [25]. In addition, the utilization of a computer-aided triage and prioritization software may have the ability to reduce hospitalization. A recent study showed the introduction of the same commercially available AI triage software into the radiological workflow was associated with a significant decrease in LoS for patients diagnosed with ICH and PE [26]. The decrease in LoS is critical in the ICH population since extended LoS has shown to increase the cost of care and risk of adverse events and medical complications [24, 25].

The reduction in mortality can also be attributed to the flagging of potentially positive scans, which can lead radiologists to prioritize the worklist and evaluate time-sensitive cases first, thus raising the index of suspicion especially in subtle or borderline findings flagged as potentially positive by the AI. Literature has shown that the prioritization of these cases also allows an earlier initiation of blood pressure control and reversal of anticoagulation or anti-aggregation drugs for the prevention of hematoma expansion in subtle ICH cases [23–25]. The AI workflow can also reduce misdiagnosis of ICH and reduce the erroneous initiation of antithrombotic treatment. Our results show a reduction of mortality in the sub-population with a history of taking anticoagulation and anti-aggregation agents after the AI implementation appears to support this clinical hypothesis.

These findings, as well as previous studies showing very high negative predictive value for AI detection of pathologies [27, 28], are pointing to the potential use of AI as a screening tool to identify patients that may need immediate medical attention in an emergency department environment, where rapid imaging diagnosis is often especially critical for patient disposition and determination of clinical management.

Currently, most radiological cases are evaluated using a first-in-first-out (FIFO) methodology rather than through a triage-optimized queuing workflow system [29]. This can lead to delayed diagnosis and treatment potentially leading to poor patient outcomes. One possible solution is to use computer-aided triage and prioritization AI solutions to evaluate abnormalities by quantifying radiological characteristics [10, 30] immediately after scan acquisition, thereby speeding up triage of the queuing workflow by flagging critical findings as they arise [29, 31], and ultimately improving patient care in clinically time-sensitive cases. Previous studies have shown a high level of accuracy and efficiency, supporting the potential effectiveness of this technology [27, 32].

### Limitations

The main limitation of this study is the fact this is a single-center, retrospective study focusing exclusively on ICH detection as well as our findings being correlative observations that may suggest, but does not prove, a causal relationship between AI implementation and decrease in mortality and morbidity decrease in patients with ICH. Future prospective studies such as a randomized clinical trial are necessary for further validation of these findings.

*In conclusion*, we have shown a correlative observation for the reduction in overall mortality and morbidity using a commercial, workflow-integrated computer-aided triage and prioritization AI solution on participants with intracranial hemorrhage. By flagging a life-threatening, time-sensitive pathology, the AI solution may improve overall reader efficiency, thus improving throughput, contributing to the timeliness in which radiologists can get to read scans with time-sensitive pathologies and possibly have the potential to enhance radiologists’ accuracy and subsequent clinical management.

### Supplementary Information

**Additional file 1: Table S1. **ICH types distribution between pre-AI and post**-**AI.

**Additional file 2: Table S2. **Number of missing data and percentage out of total in ICH, ischemic stroke and myocardial infarction datasets.

**Additional file 3: Table S3. **Hypertensive, anticoagulation, and anti-aggregant agents.

## Data Availability

The datasets generated during and/or analyzed during the current study are available from the corresponding author on reasonable request.

## Abbreviations

AI: Artificial intelligence
ICH: Intracranial hemorrhage
mRS: Modified Ranking Scale
IS: Ischemic stroke
MI: Myocardial infarction
