# Supplementary material for: The impact on clinical outcomes after 1 year of implementation of an artificial intelligence solution for the detection of intracranial hemorrhage
Source: Int J Emerg Med. 2023 Aug 11;16:50. doi: 10.1186/s12245-023-00523-y (PMC10422703; doi:10.1186/s12245-023-00523-y)
Supplement: Supplementary file 1 — Additional file 1: Table S1. ICH types distribution between pre-AI and post-AI. [file 12245_2023_523_MOESM1_ESM.docx]

|  | **ICH** | **EDH** | **SDH** | **SAH** | **IVH** |
| --- | --- | --- | --- | --- | --- |
| **Pre-AI** | 170 | 3 | 72 | 44 | 0 |
| **Post-AI** | 151 | 1 | 91 | 33 | 2 |

* p-value = 0.134
