# Supplementary material for: The impact on clinical outcomes after 1 year of implementation of an artificial intelligence solution for the detection of intracranial hemorrhage
Source: Int J Emerg Med. 2023 Aug 11;16:50. doi: 10.1186/s12245-023-00523-y (PMC10422703; doi:10.1186/s12245-023-00523-y)
Supplement: Supplementary file 2 — Additional file 2: Table S2. Number of missing data and percentage out of total in ICH, ischemic stroke and myocardial infarction datasets. [file 12245_2023_523_MOESM2_ESM.docx]

| **Missingness** | **ICH** | **Ischemic Stroke** | **Myocardial Infarction** |
| --- | --- | --- | --- |
| **White blood cells** | 6 (1.0%) | 11 (0.6%) | 3 (0.8%) |
| **Hemoglobin** | 1 (0.2%) | 9 (0.5%) | 7 (1.8%) |
| **Platelets count** | 1 (0.2%) | 9 (0.5%) | 2 (0.5%) |
