# Supplementary material for: The impact on clinical outcomes after 1 year of implementation of an artificial intelligence solution for the detection of intracranial hemorrhage
Source: Int J Emerg Med. 2023 Aug 11;16:50. doi: 10.1186/s12245-023-00523-y (PMC10422703; doi:10.1186/s12245-023-00523-y)
Supplement: Supplementary file 3 — Additional file 3: Table S3. Hypertensive, anticoagulation, and anti-aggregant agents. [file 12245_2023_523_MOESM3_ESM.docx]

| **Antiplatelet medication** | **Anticoagulant medication** | **Hypertensive medication** |
| --- | --- | --- |
| Acetylsalicylic Acid (ASA) 75-100mg | Enoxaparin sodium  20-100 mg | Doxazosin  1-4 mg |
| Prasugrel  2.5-10mg | Warfarin  1–5 mg | Hydralazine hydrochloride  10-50mg |
| Clopidogrel 75mg | Heparin | Clonidine |
|  | Dabigatran  75-150 mg |  |
|  | Apixaban |  |
|  | Rivaroxaba  4-20 mg |  |
|  | Dipyridamole  25-200 mg |  |
|  | Ticagrelor  90 mg |  |
